# Do the Child Welfare and Protection Services Involve Children in Cases With Parental Mental Health Problems? A Norwegian Case-File Study

**DOI:** 10.3389/fpsyt.2021.784022

**Published:** 2022-01-07

**Authors:** Svein Arild Vis, Camilla Lauritzen, Øivin Christiansen, Charlotte Reedtz

**Affiliations:** ^1^Regional Centre for Child and Youth Mental Health and Child Welfare, UiT-The Arctic University of Norway, Tromsø, Norway; ^2^Regional Centre for Child and Youth Mental Health and Child Welfare, Norwegian Research Centre (NORCE), Bergen, Norway

**Keywords:** parental mental illness, child involvement, child participation, child welfare and protection, concerns, COPMI

## Abstract

**Background:** Parental mental health problems is a common source of concern reported to child welfare and protection services (CWPS). In this study we explored to what extent the child was invited to participate in the investigation process. We aimed to study: (a) what was the current practice in the child protection service in Norway when the CWPS received a report of concern about children whose parents were affected by mental health problems or substance abuse, (b) to what extent were children involved and consulted, (c) which factors predicted the decision to involve the children, and (d) in cases in which conversations with children were conducted: what was the main content of the conversations.

**Method:** The study was a cross-sectional case file study (*N* = 1,123). Data were collected retrospectively from case records in 16 different child protection agencies. The cases were randomly drawn from all referrals registered in the participating agencies. Differences in how investigations were conducted in cases with and without concerns about parental mental health were analyzed using *t*-tests and chi-square testes. Predictors of child involvement in cases with parental mental health problems (*N* = 324) were estimated by logistic regression analyses.

**Results:** When the referral to the CWPS contained concerns about parental mental health, there were more consultations with parents, more frequent home visits and the investigation took longer to conclude. The children, however, were less likely to be involved. Children in such cases were consulted in 47.5% of cases. Predictors for involving the children in those cases were child age, concern about the child's emotional problems and if the child was known from previous referrals.

**Conclusion:** In Norwegian child protection investigations, in which there were concerns about the parent's mental health, conversations with children were conducted to a significantly lower degree compared to cases where the child's problem was the main concern. In such cases, the CWPS workers have to overcome a threshold before they consult with the child. The threshold decreases with child age and when case worker already knows the child.

## Introduction

Given the adverse effects of parental mental illness, there is a strong rationale for public health and preventive approaches across services, to safeguard and support the children (1, 2). The risk factors for children of parents with a mental illness (COPMI) have been thoroughly documented in studies across the world (3). In Norway it is mandatory for health care workers who treat parents with mental illness to report concerns to the child welfare and protection services (CWPS) if there is reason to believe that the child is at risk. The Norwegian Health Personnel Act further specifies that health care personnel must consult with patients who are parents, about the children's need for information or support and to provide information, guidance and direct them to relevant interventions for the family (4). Likewise, the CWPS are mandated to involve children in cases concerning their welfare and safety in accordance with age and maturity. The children's right to participate in the CWPS is established by law (The Child Welfare Act, § 1–6). These mandates are the results of increased awareness within social services and the health professional community about the potential risk for children of parents with a mental illness. Consequently, child participation is increasingly seen not only as a legal requirement in case processing but also as a mean to ensure child safety and to improve quality and effectiveness of health care and social services (5).

Despite numerous professional, political, and legislative efforts to strengthen children's participation in health and social practice, there is substantial documentation showing that child involvement is a challenge to practitioners within adult mental health care and the CWPS alike. A five-year follow-up study of identification and support for children of mentally ill parents (6) showed that even though there have been substantial efforts to change practice within adult mental health services in the past decade, children did not receive necessary support from health personnel who were treating their parents.

Intervening early and targeting adverse influences on children and parents may improve outcomes for children (7). Child involvement and child participation is a key ingredient in early intervention. Additionally, psychoeducation is a common component across programs for parents with mental illnesses and their children (8). In the context of parental mental illness, psychoeducation is seen as a tool to reduce feelings of guilt and shame from materializing in the children and their parents. A lack of openness about mental illness is also thought to restrain children from venting emotions such as anger, despair and insecurities about their own life situation and that of their parents. Subsequently, when there is a mental illness in the family, children need accurate mental health information (9, 10). Not receiving information and support may severely affect the lives of these children. Faugli et al. found that children who sought information were often ignored by the health personnel (11).

There is substantial documentation showing that establishing a dialogue with children is a challenge to many adult helpers. Many of the barriers to child involvement seem to be the same across service settings, such as the professional's attitudes and skills (12).

Child welfare and protection workers strive to balance children's right to participate on the one hand, and the right to protection on the other hand. This is especially the case when the case concerns adult's problems, such as parent conflicts, mental health issues, and substance abuse. The workers are worried that they will expose children to such problems because it may be a burden or even harmful to them, which may be avoided by not involving them (13, 14). Age may be another important factor. A study carried out among Norwegian CWPS workers found that the most experienced workers were also the most reluctant to let children participate in child protection processing (15). Other significant explanations for the reluctance to involve children are social workers' and health personnel's lack of professional confidence, skills and tools (14, 16, 17). Previous studies have pointed out that the adult mental health services regarded their competence and knowledge about support for the children of their patients as limited, and that they considered the CWPS to be a more suited service to provide for the children's needs (18). Furthermore, the results showed that adult mental health workers lacked skills in how to approach the family, how to develop trust and confidence, and how to discuss negative consequences of the parental mental illness for the children. Additionally, many reported that they lacked the competence to assess the needs children may have and explained this by their educational background not being child specific (19). On the other hand, little is known about how the CWPS addresses cases of parental mental health problems. We therefore do not know if the CWPS involve the children of parents where there is a reported concern about mental health issues. Studying the CWPS approach to these children may inform us about important issues to be aware of in the overall approach to support COPMI.

## Aims

The main objective of the current study was to explore the child welfare and protection services' approaches to families affected by parental mental illness. Admittedly, child welfare legislation does differ between countries, and some aspects of professional practice may be specific to certain contexts. However, as illustrated by the introductory review there are also aspects of professional practice that is rooted in conceptions about children's abilities and vulnerabilities which transcends borders and traditions. We therefore believe that studying if families where there are concerns about parental mental health are approached differently than families with other types of concerns, is important. The aims of the current study were therefore: (a) to identify who the CWPS in Norway consulted when they received a report of concern about children whose parents are affected by mental health problems, (b) to what extent children were involved and consulted, (c) which factors predicted the decision to involve the children, and (d) in cases where conversations with children were conducted: what was the main content of the conversations.

## Method

The study is part of a large national research project that was initiated in 2017. The project was approved by the Council for Duty of Confidentiality and the Norwegian Center for Research Data. The researchers were given access to social work records by a decision from the Directorate for Children, and Family Affairs in Norway. This decision allowed the researchers to extract data from case files without seeking informed consent. A license for handling and storage of data were granted by the Norwegian Data Protection Authority on the 29.06.2017 (reference number: 7/00411-2/CDG).

### Design and Procedures

The study was designed as a case file study which was carried out retrospectively. A total of 1,365 child welfare and protection cases were randomly drawn from all referrals registered in 16 participating agencies in the period of January 2015 to December 2017. The number of cases from each agency varied between 50 and 150 depending on the size of the agency. The reason why we sampled agencies by size is that we wanted the number of cases drawn from each agency to be about the same proportion of the total available sample from that agency. Data were collected and coded from case records. The researchers were given access to the casefiles and to electronic systems for recordkeeping by the CPS agency. All case files were coded on site at the agency by the use of an electronic web-based data entry form that was developed specifically for this purpose. The data entry form was developed and tested for interrater reliability by independent coding of 20 cases by two researchers. The results showed an average interrater agreement of 86.9%. A total of 13 variables had low reliability (<80% interrater agreement). Three of those were eliminated from the form because it was concluded that reliable information could not be obtained. The remaining 10 variables were reformulated, and the coding manual was revised with better explanation of codes. After this revision the reliability of the instrument was re-tested by independent coding of 42 cases by two researchers. At this second step, interrater agreement was 90.8%. In health research, an interrater agreement over 80% generally are considered acceptable (20). The variables and the codes from the form is available from the corresponding author upon request.

### Participants

For the current analyses we included all the cases that were screened in for a child protection investigation (*N* = 1,123). Fifty-three percentage of the sample were boys and the mean age was 8.9 years (SD = 5.1). In a total of 41.6 % of the referrals, the family had immigrant background. Immigrant background was defined as the child or one of the parents being born in a country other than Norway.

### Measures

Referrals to CWPS in Norway is most usually a free text letter submitted by a concerned third party. We coded the concerns in the referral letter as present or absent because this is all that safely can be concluded with high level of reliability. The following types of concerns was coded as present or absent in the referral (i) parental mental health problems or substance abuse problems (ii) child developmental problems, (iii) child externalizing behavior problems (iv) child emotional problems. The main characteristics of the investigation process was registered. This included counting (i) the duration of the investigation measured in number of days before the investigation was concluded, (ii) number of meetings between CWPS and parents, (iii) number of home visitations by the CWPS and (iv) if additional information had been requested from health care services, school, police, social services or other CWPS agencies. Whether or not there had been a consultation with the child as part of the investigation was registered. In instances in which such a consultation had taken place (*N* = 680) the main content of the consultation was coded into seven different pre-determined content Those were (i) exploratory conversation about conditions at home, (ii) information sharing, (iii) conversation to obtain child's opinions, (iv) investigative conversation about episode in the family, (v) supportive conversation, (vi) general conversation without specific aim, (vii) no information about the content. These categories were developed by the researchers based upon the theory of general procedures for the participation of children (21).

### Statistical Analyses

Bi-variate differences in the main characteristics of the investigation process, with and without concerns about parental mental health problems were analyzed using *t*-tests for continuous variables and chi-square testes for categorical variables. Predictors for child consultations in cases with parental mental health problems (*N* = 324) were examined in multivariate analysis using binary logistic regression. In the regression analysis all predictors were entered at together.

## Results

Our first aim was to identify who CWPS in Norway consulted in the investigation when they received a report of concern about children whose parents are affected by mental health problems. When receiving a report of concern, the CWPS investigations most commonly consisted of consultations with the child, consultation with a parent, and home visits. In addition, the CWPS obtained information from other services such as health care, school/kindergarten, police, and other social services. We investigated if there were differences in how CWPS investigations were conducted in cases reported with parental mental health issues compared to cases in which such problems were not reported. The results showed that when the report to the CWPS contained concerns about parental mental health there were more consultations with parents, more frequent home visits and the investigation took longer to conclude. The children however were less likely to be consulted. On average children were consulted in 47.5% of those cases (Table 1).

**Table 1:** CWPS investigations in cases referred for parental mental health problems and/or parental substance abuse vs. other problems (*N* = 1,059–1,123).

**Investigation activity**	**Mental health/substance abuse problem in family** ***N*** **(%)**	**Other problem** ***N*** **(%)**	**χ^2^ (df)**
Consultations with the child = yes	154 (47.5%)	526 (65.8%)	32.3 (1)^***^
Information from health care = yes	232 (71.6%)	485 (60.7%)	11.9 (1)^**^
Information from school/child care = yes	177 (54.6%)	549 (68.7%)	20.0 (1)^***^
Information from police = yes	111 (34.3%)	253 (31.7%)	0.71 (1)
Information from social services = yes	78 (24.1%)	117 (14.6%)	14.3 (1)^***^
Information from other CPS agency = yes	21 (6.5%)	52 (6.5%)	0.0001 (1)
	**M (SD)**	**M (SD)**	**t (df)**
Duration of the investigation (days)	72.6 (58.6)	64.6 (53.9)	−2.19 (1118)^*^
Consultations with a parent (number of times)	3.18 (2.85)	2.77 (2.08)	−2.34 (469^a^)^*^
Home visits (number of times)	0.95 (1.44)	0.70 (0.85)	−2.90 (418^a^)^**^

* *p < 0.05*,

** *p < 0.01*,

*** *p < 0.001*.

a *Adjusted for non-equal variance*.

We also investigated which other child and case characteristics that may explain whether children were consulted in cases with referrals of suspected parental mental health problems. In the multivariable analysis we identified three statistically significant predictors. The first was child age. For a 5-year difference in child age, the odds ratio for a child consultation were 3.18. This means that a 12-year-old child were more about three times more likely to be consulted than a 7-year-old child. The second predictor was if a concern about the child's emotional problems had been raised in the report. Then the child was about 2.8 times more likely to be consulted. The third predictor was if the child was known by the agency from previous reports, then the chance of a child consultation was increased by a magnitude of about 1.3 for each previous report (Table 2).

**Table 2:** Predictors for child consultation in CWPS cases with parental mental health problems and/or parental substance abuse problems (*N* = 324).

	**B**	**OR (95% CI)**
Number of previous referrals	0.23	1.26 (1.07–1.48)^**^
Child sex = male	0.38	1.46 (0.86–2.47)
Child age	0.23	1.25 (1.18–1.33)^***^
Child immigrant background = no	0.03	1.03 (0.56–1.87)
Concern about child developmental problem = no	−0.06	0.94 (0.24–3.78)
Concern about child externalizing problems = no	−0.01	1.0 (0.42–2.36)
Concern about child emotional problem = no	1.02	2.76 (1.004–7.58)^*^

* *p < 0.05*,

** *p < 0.01*,

*** *p < 0.001*.

A final aim was to study the cases where children were consulted and identify what the content of conversations with children was. The most common form of conversation in our sample was exploratory conversations about conditions in the home (72.6%). In such conversations, the child was encouraged to talk about how it is at home without it being related to episodes or specific events. In this category, there may also be exploratory conversations about the child's everyday life, for example how the child is doing at school.

Furthermore, 36.9% of the child conversations took the form of an informative conversation. An informative conversation is characterized by the child receiving information about the case and/or what will happen in the future. In 31.5% of the cases in our sample, the focus in the children's conversation was on obtaining the child's point of view or opinions. This was more frequently the topic in cases with suspected parental mental health problems. More frequent in those types of cases were also consultations which had a supportive rather than an investigatory purpose (Table 3).

**Table 3:** Differences in content of CWPS conversations with children (*N* = 680).

**Content**	**Referrals with mental health**		**Referrals with other**		**X^**2**^ (df)**
	**problems** **(***n*** = 154)**		**problems** **(***n*** = 526)**		***P*** **for the difference**
	***n***	**%**	***n***	**%**	
Exploratory conversation about conditions at home	114	71.1	374	71.1	0.50 (1)
Informational conversation	51	33.1	199	37.8	1.14 (1)
Conversation to obtain child's opinions	61	39.6	152	28.9	6.35 (1)^*^
Investigative conversation about episode in family	20	13.0	126	24.0	8.50 (1)^**^
Supportive conversation	17	11.0	31	5.9	4.81 (1)*
General conversation without specific aim	8	5.2	31	5.9	0.11 (1)
No information about the content	19	12.3	34	6.5	5.72 (1)^*^

* *p < 0.05*,

** *p < 0.01*.

## Discussion

The results showed that when the CWPS initiated an investigation based on a report of concern about parental mental health, the investigation was significantly more extensive in time and efforts compared to investigations of other types of concerns. In particular, the CWPS workers carried out more consultations with parents and more frequently made home visits. A reasonable interpretation of these findings is that such reports were considered more serious by the CWPS and hence that they conduct more thorough investigations. Another interpretation is that the CWPS workers, who are social workers and not health personnel, feel less competent and more insecure about how to evaluate the seriousness of mental health problems. This assumption might explain why these investigations became more extensive with respect to time spent and the number of consultations with parents. Parents' fear of the CWPS and subsequent resistance to inform CWPS workers about parental mental illness may also result in more complicated and time-consuming investigations compared to other types of problems. Establishing a trusting and cooperative relation with the parents, particularly when there are concerns about alcohol and/or substance abuse can be more difficult and time consuming.

When examining to what extent children were involved in cases of parental mental illness, we found that the child was less involved when the report concerned parental mental health or substance abuse. It is hard to understand what explains this practice, especially since the CWPS workers spend more time investigating these cases compared to other cases. However, this finding resonates with previous research which shows that one of the most important reason why caseworkers do not talk to children about difficult topics is that the belief that it may be a burden for children to become involved in adults' problems (14). There is nevertheless no reason to believe that resistance to talking to children in such situations is specific only to the child welfare and protection services. The same belief has been identified among health personnel (18).

In terms of which factors may predict the decision to consult the children, we found that child consultations were more likely to take place with increasing child age. Age is however not solely a predictor for involvement in cases with reported parental mental health concerns, but in all cases within the CWPS. This finding is an expected one, in line with many other studies (22–24). The main reason for this is that children are increasingly able, and perhaps also willing, to talk about family problems as they mature, or that the CWPS considers them to be less vulnerable compared to younger children and therefore more frequently initiate consultations. Although child age should not automatically disqualify children from an opportunity to talk to the social worker, we do recognize that there are limits to what can reasonably be expected from the youngest children. However, as younger children are more dependent of developmental and social support from their parents, they are also more vulnerable to lack of proper care, and hence social workers should acknowledge this in their work with younger children.

Another result of interest is that previous referrals increased the likelihood of children being involved. One plausible explanation may be that when there are previous referrals the CWPS already has knowledge about and may be acquainted with the family and the child. When the caseworker already has established a relationship with the child, this may contribute to reduce the fear that reaching out to the child will be disruptive or harmful for the child. It is known from previous studies (25) that many previous referrals are used as an indication that there is increased risk of child abuse or neglect. Increased perception of risk for the child due to the conditions at home may offset the fear a case worker has of disrupting the child by consultations.

There are good reasons to consult with children when there are concerns about the child's emotional problems. First and foremost because internalized mental health problems cannot readily be assessed without the contribution from the person in question, some form of self-report is usually required and recommended (26).

In terms of identifying what the main content of the conversations with children were when such conversations had been conducted, we found that nearly two-fifths of the conversations were aimed at giving the child information about the ongoing investigation. Usually, the social worker will have to explain to the child what the reason for the investigation is and thus disclose some information about the parents' problems in order to initiate a conversation with the child. As discussed above reluctancy to disclose such information may explain why consultations are less likely to occur. However, when they do occur this provides an opportunity to not only seek information from the child but also to provide some basic psycho-educative support. This is of great importance given the high risk these children have for developing mental health issues themselves (1, 27–29). This is particularly important when we take into consideration the relatively high chance that the case will ultimately be dismissed without any further service provision for the child or the family (28). It is positive that conversations with an aim to support the child were more often recorded in cases with reported parental mental health problems, albeit the frequency of this types of conversations were overall very low. We are quite certain that more than 11 % of the children in such cases are in need of support given the high prevalence of mental health issues among COPMI (30, 31). Admittedly, for practical and legal reasons there are limitations to how comprehensive support measures can be at this stage in CWPS case processing. However, as a minimum it could be expected that (i) the situation is explained to the child with emphasis upon the reason for contact with the CWPS, (ii) that the child is informed about what is going to happen and eventually that (iii) the reasons for subsequent decision are clearly explained. Interview studies have indicated that this is expected by children (32).

In overall 31.5% of the cases in our sample, the focus in the conversations with the child was on obtaining the child's point of view or opinions. In relation to children's right to be heard, this may seem to be a somewhat low number. The child's point of wiew was, however, more often part of the conversation if the concern was about parental mental health (39.6%). It is possible that the CWPS has talked to the child about the child's opinions and wishes without recording it. Nevertheless, the child's voice and what the child thinks about the case should emerge in a larger proportion of cases. We therefore call upon all professional partners to collaborate and to keep pushing the participation agenda forward. It is our belief that it is helpful for the development and health of COPMI children.

## Implications For Practice

The shortcomings in current practice in terms of involving children in cases where a parent has mental health problems have not previously been documented. However, previous studies in Norway have shown how the CWPS investigates their cases and their process from concern to decision-making (33). It was concluded that there is a need for a quality system to achieve quality assurance in practice. Studies have documented that the professionals would prefer to have more guidance and a framework to assist the assessment of risk (28). Furthermore, research has shown that the way investigations are carried out also differs between agencies (28). A national knowledge-based system and focus on the child's needs, can contribute to better documentation and a CWPS practice that to a larger extent involves the children. In January 2022, several changes will be made to the Norwegian Child Welfare Act. The intention is to strengthen prevention of child maltreatment. Children's right to participate will also be strengthened. It is, however, unclear how the amendments will be implemented in practice. There is no reason to believe that amendments in legislation will take place without an operationalized system to support a new practice. The findings of this study highlight the need for national guidelines that makes it mandatory to include children in all child protection cases.

## Limitations And Strengths

This analysis is based upon what was recorded in case files. Not everything a social worker does during case processing goes into written records. It is therefore possible and likely that the numbers presented here slightly underestimates the extent and type of contact between social workers and children. That being said, the findings do not deviate substantially from what has been reported by others. The case files included in this study were solely from child welfare and protection services, and not from adult mental health services. Subsequently, we do not have information about the parents' diagnosis. We do not know if the type of mental health problems the parent had may have influenced the decision to not involve children in some cases.

It is a substantial strength that our data were randomly drawn and represents a large and representative sample.

## Conclusions

In child protection cases in which the concern is mainly about the parent's mental health or substance abuse, conversations with children are conducted to a significantly lower degree compared to other cases. The CWPS are more likely to consult with older children and if the child has been referred before. The findings indicates that social workers perceptions about child vulnerability is a major obstacle for child inclusion and participation in child protection investigations. More children should be consulted in cases with reported concerns about parental mental health. Knowing that a substantial proportion of these children have or will develop problems themselves we cannot maintain a high threshold for consulting them. In our view, a child consultation should not only seek to extract information from children but should also seek to utilize the potential preventive effects that lies in basic psychoeducation. The CWPS workers are in the best position to make sure the child is involved and receives information. Child involvement is a goal that can be achieved.

## Data Availability Statement

The datasets presented in this article are not readily available because our license from the data protection authority does not allow sharing of raw data. Requests to access the datasets should be directed to Svein Arild Vis, svein.arild.vis@uit.no.

## Ethics Statement

The studies involving human participants were reviewed and approved by Norwegian Data Protection Authority NSD-Norwegian center for research data. Written informed consent from the participants' legal guardian/next of kin was not required to participate in this study in accordance with the national legislation and the institutional requirements.

## Author Contributions

SV designed the study and conducted the analyses. SV, ØC, and CL collected the data. SV, CL, ØC, and CR contributed to the writing of the article manuscript. All authors agree to be accountable for the content of the work.

## Funding

The study was funded by the Norwegian Directorate for Children, Youth and Family Affairs (Bufdir) and UiT–The Arctic University of Norway.

## Conflict of Interest

The authors declare that the research was conducted in the absence of any commercial or financial relationships that could be construed as a potential conflict of interest.

## Publisher's Note

All claims expressed in this article are solely those of the authors and do not necessarily represent those of their affiliated organizations, or those of the publisher, the editors and the reviewers. Any product that may be evaluated in this article, or claim that may be made by its manufacturer, is not guaranteed or endorsed by the publisher.

## Nomenclature

COPMI: Children of parents with a mental illness
CWPS: Child Welfare and Protection Services
CRC: Convention of the Rights of the Child
